# Detection of Suicide Risk Using Vocal Characteristics: Systematic Review

**DOI:** 10.2196/42386

**Published:** 2022-12-22

**Authors:** Ravi Iyer, Denny Meyer

**Affiliations:** 1 Centre for Mental Health Swinburne University of Technology Hawthorn Australia

**Keywords:** voice, suicide, suicidal, biological signal processing, machine learning, systematic review, review methodology, risk, speech, mental health

## Abstract

**Background:**

In an age when telehealth services are increasingly being used for forward triage, there is a need for accurate suicide risk detection. Vocal characteristics analyzed using artificial intelligence are now proving capable of detecting suicide risk with accuracies superior to traditional survey-based approaches, suggesting an efficient and economical approach to ensuring ongoing patient safety.

**Objective:**

This systematic review aimed to identify which vocal characteristics perform best at differentiating between patients with an elevated risk of suicide in comparison with other cohorts and identify the methodological specifications of the systems used to derive each feature and the accuracies of classification that result.

**Methods:**

A search of MEDLINE via Ovid, Scopus, Computers and Applied Science Complete, CADTH, Web of Science, ProQuest Dissertations and Theses A&I, Australian Policy Online, and Mednar was conducted between 1995 and 2020 and updated in 2021. The inclusion criteria were human participants with no language, age, or setting restrictions applied; randomized controlled studies, observational cohort studies, and theses; studies that used some measure of vocal quality; and individuals assessed as being at high risk of suicide compared with other individuals at lower risk using a validated measure of suicide risk. Risk of bias was assessed using the Risk of Bias in Non-randomized Studies tool. A random-effects model meta-analysis was used wherever mean measures of vocal quality were reported.

**Results:**

The search yielded 1074 unique citations, of which 30 (2.79%) were screened via full text. A total of 21 studies involving 1734 participants met all inclusion criteria. Most studies (15/21, 71%) sourced participants via either the Vanderbilt II database of recordings (8/21, 38%) or the Silverman and Silverman perceptual study recording database (7/21, 33%). Candidate vocal characteristics that performed best at differentiating between high risk of suicide and comparison cohorts included timing patterns of speech (median accuracy 95%), power spectral density sub-bands (median accuracy 90.3%), and mel-frequency cepstral coefficients (median accuracy 80%). A random-effects meta-analysis was used to compare 22 characteristics nested within 14% (3/21) of the studies, which demonstrated significant standardized mean differences for frequencies within the first and second formants (standardized mean difference ranged between −1.07 and −2.56) and jitter values (standardized mean difference=1.47). In 43% (9/21) of the studies, risk of bias was assessed as moderate, whereas in the remaining studies (12/21, 57%), the risk of bias was assessed as high.

**Conclusions:**

Although several key methodological issues prevailed among the studies reviewed, there is promise in the use of vocal characteristics to detect elevations in suicide risk, particularly in novel settings such as telehealth or conversational agents.

**Trial Registration:**

PROSPERO International Prospective Register of Systematic Reviews CRD420200167413; https://www.crd.york.ac.uk/prospero/display_record.php?ID=CRD42020167413

## Introduction

### Background

Telehealth alternatives may soon replace in-person visits to providers of primary health care [1]. Telehealth is effective in reducing the severity of mental illness [2], leading the Australian government to commit to universal access to telehealth care alternatives [3].

The potential utility of telehealth services to the community is undeniable, being ideally suited to reach sectors of the population that have historically faced barriers to access. In particular, rural and remote communities face unique challenges in terms of both economic disparity and location [4]. Telehealth services are also appealing to health care consumers of a younger age (19-44 years) [5]. These cohorts substantially overlap with those most at risk of suicide [6,7].

Telehealth is also being used in other, more novel ways. Primary health care providers in the United States are increasingly using *forward triage*, where patients are assessed before arrival via telehealth means and often via a conversational agent [8]. However, this may prove challenging when mental health is the main presenting issue as suicidality is a feature of most mental health disorders [9]. Thus, the transition from in-person provision of health care raises important ethical considerations. For example, how can escalation in suicide risk be accurately and efficiently assessed in the absence of in-person cues?

In >50 years of research, traditional methods of suicide risk assessment (ie, surveys) have yielded little more than chance accuracy in identifying elevated suicide risk [10]. Franklin et al [10] suggested that suicide risk assessment would benefit from the use of risk algorithms that can assess multiple predictors simultaneously. However, they did not consider the use of biological markers in their review. Such markers do not rely on patient testimony and may prove more accurate in the assessment of suicide risk [11].

Suicide-related biological marker research has focused mainly on identifying neurobiological changes associated with elevated risk. However, the downstream effects of these neurobiological changes may also be apparent and remain underresearched. In particular, changes in speech production and articulation—the subject of this review—have been associated with elevated suicide risk, as indicated in this section. There is an identified need to leverage these novel technologies in the provision of real-time adaptive personalization of counseling content to match consumer emotions [12]. This is consistent with the recent recommendations of Balcombe and De Leo [13], who also argue for the real-time tracking of consumer emotions via machine learning–trained predictive models that can assist in delivering more timely and efficient mental health care support at scale.

In their review of vocal characteristics used to identify suicide risk, Cummins et al [14] found that many characteristics could prove viable in the detection and differentiation of suicide risk presentations. They identified 4 types of vocal characteristics used for this purpose: prosodic (long-term changes in rhythm, stress, and intonation), voice production, formant (changes in vocal tract properties), and frequency (pitch). Cummins et al [14] noted that the speech of individuals at high risk of suicide is often distinguished by a hollow, toneless, and monotonous quality or by a breathy tone, which corresponds to a marked change in spectral slope (accuracies of 90% when using this variable to predict suicide risk) [15]. Cummins et al [14] also noted that the second formant bandwidth and power spectral densities between 0 and 1000 Hz are promising candidates for further research (accuracies of 90% were obtained using a combination of these features).

Homan et al [16] recently reviewed the use of both voice signals and text-based data to predict suicide risk. The findings of Cummins et al [14] were supported by those of Homan et al [16], who also suggested pause length and jitter (the timing of the glottal pulse) as additional candidates. However, the authors did not discuss accuracy of prediction or the methodological specifications informing the systems of classification used. However, both Cummins et al [14] and Homan et al [16] agree that, despite prevailing methodological issues, namely, small sample sizes, lack of control of covariates, and validity of ground truth, there is evidence that suicide risk does alter the human voice in substantive and important ways and may be predictive of elevated suicide risk. Other authors have also noted the equivocality of current findings and the need for further confirmatory research [17].

### Objectives

Thus, the primary objective of this systematic review was to assess the accuracy of vocal characteristics in differentiating between individuals at risk of suicide and those who are not at risk. The secondary objective was to assess the methodological specifications used in these systems of classification.

## Methods

### Design

**Figure 1:**
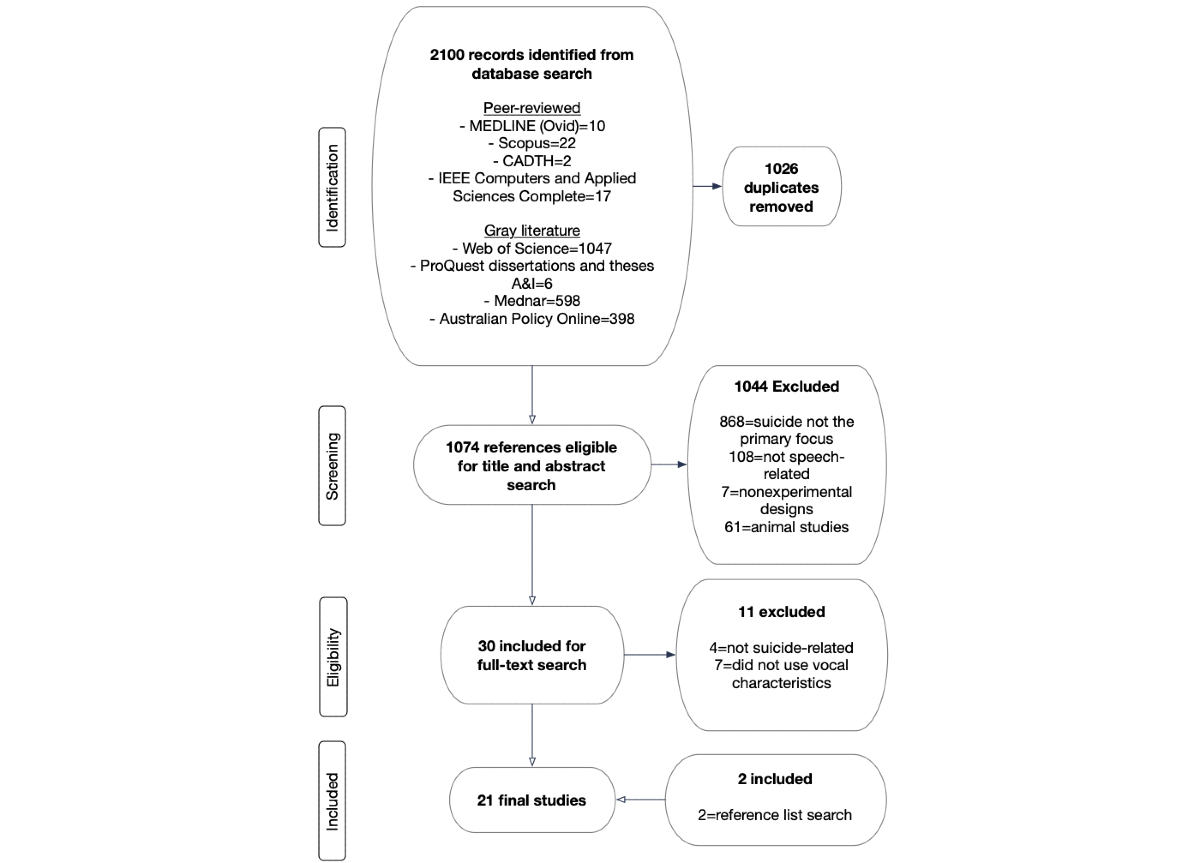
PRISMA (Preferred Reporting Items for Systematic Reviews and Meta-Analyses) flow diagram.

This systematic review was conducted using the PRISMA (Preferred Reporting Items for Systematic Reviews and Meta-Analyses) standards [18] (Figure 1) and checklist (Multimedia Appendix 1). The systematic review protocol was registered with PROSPERO on April 28, 2020 (registration CRD420200167413) [19]. The Population, Intervention, Comparator, Outcome, and Study Design framework defined the research questions and search terms. The research questions were as follows: Which vocal characteristics can differentiate between high and low risk of suicide among both adult and adolescent populations with a high level of accuracy? and What are the methodological specifications used to derive the vocal characteristics and inform the levels of accuracy obtained?

### Information Sources

MEDLINE via Ovid, Scopus, Computers and Applied Sciences Complete, and CADTH, in addition to the gray literature databases Web of Science, ProQuest Dissertations and Theses A&I, Australian Policy Online, and Mednar, were searched initially from January 1, 1990, to December 31, 2020, and updated in January 2022.

### Search Strategy

Search strategies were developed using Medical Subject Headings and keyword string searches that included synonyms of “suicide,” “vocal,” and “algorithm” as separate blocks. A final block of various vocal characteristics was also added, informed by a preliminary survey of the literature. Gray literature was included to ensure a breadth of sources and that insights from unpublished resources might also be included (ie, theses). As a final step, the reference lists of all the included studies were reviewed to ensure that all possible studies were included. Refer to Multimedia Appendix 2 for all terms and search strategies used.

### Inclusion and Exclusion Criteria

The participants were human, with no language or age restrictions applied. The focus of inquiry was single or multiple measures of vocal quality, which included measures of volume, pace, pitch, rate, rhythm, fluency, articulation, enunciation, and tone. The presence of suicidal ideation or recent behavior was considered the intervention, whereas the absence of such ideation or behavior was the comparator. The primary outcome was a validated measure of suicide risk, whereas no setting restrictions were applied. The study design included randomized controlled trials, cohort studies only, and other unpublished research (ie, theses).

We followed the International Classification of Diseases, 11th Revision, which defines *suicidal ideation* as thoughts, ideas, or ruminations about the possibility of ending one’s life; *suicide behavior* as concrete actions that are taken in preparation for fulfilling a wish to end one’s life; and *suicide attempt* as a specific episode of self-harming behavior undertaken with the conscious intention of ending one’s life.

Studies were excluded when they involved animal populations; were unrelated to either the evaluation of vocal quality or suicide risk; did not involve a comparison group; were single-case studies; or did not provide sufficient detail to establish all the Population, Intervention, Comparator, Outcome, and Study Design criteria.

### Selection and Data Collection Process

Both authors independently reviewed the title, abstract (step 1), and full text (step 2) of each publication identified in accordance with the inclusion and exclusion criteria. NVivo (version 12; QSR International) [20] was used to classify each publication for inclusion (green), in doubt (amber), and exclusion (red), with any in-doubt publications discussed further by the authors before consensus. Each publication was also coded to provide a rationale for exclusion (ie, 1=suicide not the primary focus, 2=non–speech-related, 3=animal study, and 4=no comparison between groups).

### Data Extraction and Quality Assessment

Information was extracted from the included studies according to the following five categories: (1) participant recruitment and characteristics, (2) preprocessing methodological considerations, (3) vocal characteristics, (4) accuracy, and (5) algorithmic approach to classification.

The included studies were also assessed for quality of evidence by RI, confirmed by DM using the Oxford Centre for Evidence-Based Medicine Levels of Evidence. Each study was rated with a score of 1 to 5, where randomized controlled trials typically scored higher (score=2) than nonrandomized studies (score=3). Disagreements were resolved through discussion.

### Risk-of-Bias Assessment

RI assessed the methodological quality of the final studies using the Risk of Bias in Non-randomized Studies tool developed by the Cochrane Collaboration [21]. The Risk of Bias in Non-randomized Studies tool involves 3 stages of assessment, including specification of the research question (stage 1) and specification of the effect of interest, result to assess, identification of confounders and cointerventions, risk-of-bias judgment for each domain, and an overall risk-of-bias determination for each study (stage 2). This is then synthesized as an overall risk-of-bias assessment for all studies (stage 3). The risk-of-bias domains include confounding, selection of participants, classification of interventions, deviations from planned interventions, missing data, outcome measurement, reporting of results, and overall bias. The risk of bias was assessed as low, moderate, or high. The risk-of-bias assessments are available in Multimedia Appendix 3 [15,22-41].

### Synthesis Methods

The included studies were heterogeneous in terms of the vocal characteristics assessed and reporting, with classification accuracies included in some studies and mean outcome measures included in others. A narrative synthesis was used to organize the information from the included studies where mean outcome measures were not reported. The guidelines of Rodgers et al [42] were applied, which included a preliminary analysis and exploration of relationships followed by the assessment of the robustness of the synthesis.

Wherever possible, data were presented in tabular form, with information broadly organized around study and participant characteristics, followed by the 2 study questions: classification accuracy of suicide risk using vocal characteristics in the first section and methodological steps taken in the second section.

Where mean outcome measures were reported, a random-effects model meta-analysis was conducted to synthesize the available information, although multiple vocal characteristics were typically reported in a small number of studies. Using the R package *metafor* (version 3.8-1; R Foundation for Statistical Computing), standardized mean differences were derived from the mean outcome measures reported. Standardized mean differences for each vocal characteristic were then illustrated using a forest plot. All data used in this systematic review are available in Multimedia Appendix 4.

## Results

Figure 1 illustrates the number of studies that were initially identified, screened, deemed eligible, and included in the final analysis.

### Summary of the Included Studies

**Figure 2:**
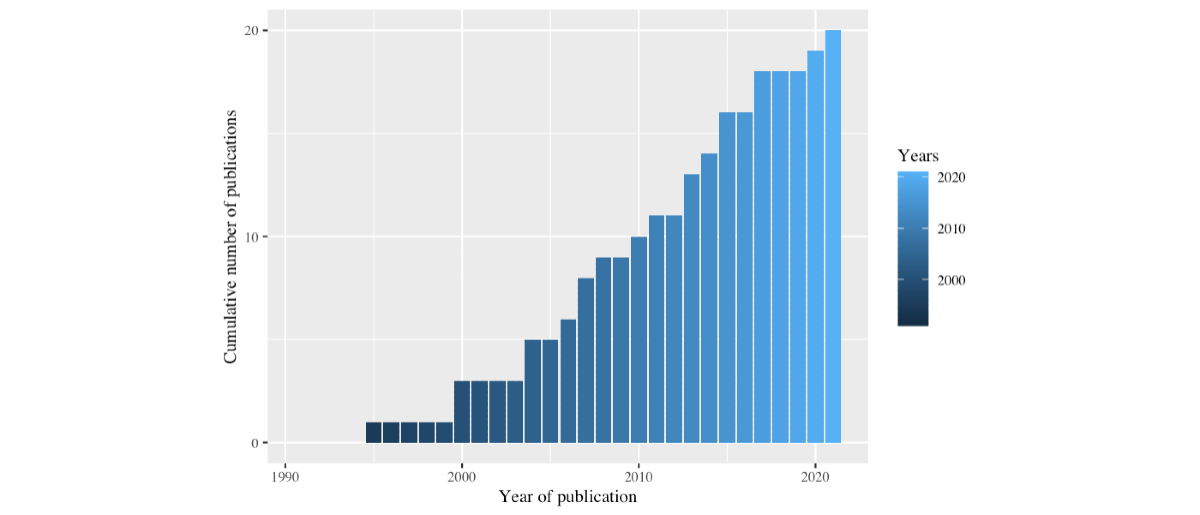
Cumulative number of publications by year.

A total of 1074 studies were initially identified. After careful screening, 21 studies from 4 countries were found to comply with all inclusion and exclusion criteria. These studies are summarized in Table 1. The included studies featured 1734 participants overall, with 14% (3/21) of the studies [15,22,23] involving adolescent populations only. The publications by Campbell [24], Sanadi [25], and Sinha [26] were theses, whereas the remaining studies (18/21, 86%) were peer-reviewed journal articles. Most studies (11/21, 52%) were observational in nature, and most studies used participant recordings from either the Vanderbilt II database (8/21, 38%) or the Silverman and Silverman perceptual study (7/21, 33%). These data sources are summarized in Table 2. The number of studies published by year is illustrated in Figure 2 and can be seen to increase slightly from 2006 onward.

**Table 1:** Sample characteristics of the included studies (N=21).

Author, year (country)	Participants, N	Design	Sample	Assessment measure	Participant age (years)
Anunvrapong and Yingthawornthuk [27], 2014 (Thailand)	30 female	Observational	Psychiatric inpatients	Psychiatric interview	—^a^
Belouali et al [28], 2021 (United States)	124	Longitudinal	Veterans	Patient Health Questionnaire-9	—
Campbell [24], 1995 (United States)	3	Observational	Telephone call recordings	Clinician-rated	—
Figueroa Saavedra et al [29], 2020 (Chile)	100 (60 female and 40 male)	Cross-sectional	University students	Okasha Suicidality Scale	18-19
France et al [30], 2000 (United States)	115 (38 female and 77 male)	Observational	Psychiatric inpatients	Beck Depression Inventory and Hamilton Depression Rating Scale	25-65
Keskinpala et al [31], 2007 (United States)	169 (92 female and 77 male)	Observational	Psychiatric inpatients	Clinician-rated	—
Nik Hashim et al [32], 2015 (Malaysia)	89 (54 female and 35 male)	Observational	Psychiatric inpatients	Beck Depression Inventory-II, Hamilton Depression Rating Scale, Mini International Neuropsychiatric Interview, and Pierce Suicide Intent Scale	—
Nik Hashim et al [33], 2015 (Malaysia)	126	Controlled study	Psychiatric inpatients	Hamilton Depression Rating Scale	22-62 (mean 42.6, SD 10.2)
Ozdas et al [34], 2000 (United States)	20 male	Controlled study	Psychiatric inpatients	Hamilton Depression Rating Scale	25-65
Ozdas et al [35], 2004 (United States)	30 male	Controlled study	Psychiatric inpatients	Clinician-rated	25-65
Ozdas et al [36], 2004 (United States)	30 male	Controlled study	Psychiatric inpatients	Clinician-rated	25-65
Pestian et al [37], 2017 (United States)	379	Controlled study	Psychiatric inpatients or outpatients	Clinician-rated	Adolescent
Sanadi [25], 2011 (United States)	60	Observational	Psychiatric inpatients	Clinician-rated	—
Scherer et al [22], 2013 (United States)	381	Controlled	Databases of recordings	Patient Health Questionnaire-9 and Beck Depression Inventory	Adult (mean 44.7, SD 12.37) and adolescent (13-17)
Scherer et al [15], 2015 (United States)	60	Controlled	Psychiatric inpatients	Columbia Suicide Severity Rating Scale, Suicidal Ideation Questionnaire-Junior, and Ubiquitous Questionnaire	13-17
Sinha [26], 2013 (United States)	17	Observational	Psychiatric inpatients	Clinician-rated	25-65
Subari et al [38], 2010 (Malaysia)	30	Observational	Psychiatric inpatients	Clinician-rated	25-65
Venek et al [23], 2017 (United States)	60	Controlled	Psychiatric inpatients	Columbia Suicide Severity Rating Scale, Suicidal Ideation Questionnaire-Junior, and Ubiquitous Questionnaire	13-17 (mean 15.47, SD 1.5)
Yingthawornsuk et al [39], 2006 (United States)	32 male	Observational	Psychiatric inpatients	Beck Depression Inventory-II	25-65
Yingthawornsuk et al [40], 2007 (United States)	20 female	Observational	Psychiatric inpatients	Beck Depression Inventory-II	25-65
Yingthawornsuk and Shiavi [41], 2008 (Thailand)	25 male	Observational	Psychiatric inpatients	Beck Depression Inventory-II	25-65

^a^Not available.

**Table 2:** Sources of study participants.

Participant source, year	Details	Studies
Vanderbilt II database, 1993 [43]	Database of recordings of interviews with individuals responding to an advertisement for low-cost psychotherapy; participants met DSM-IV^a^ criteria for major depression	Anunvrapong and Yingthawornthuk [27] France et al [30] Ozdas et al [34-36] Subari et al [38]
Cognitive behavioral therapy and psychopharmacology study, 1992 [44]	Database of recordings of psychotherapy sessions comparing the effects of cognitive behavioral therapy with psychopharmacological interventions	France et al [30] Ozdas et al [34,36]
Silverman and Silverman perceptual study, nd^b^ [45]	Database of recordings of psychotherapy sessions and suicide notes of patients who had attempted or completed suicide within hours to weeks of the recordings	France et al [30] Ozdas et al [34-36] Subari et al [38]
Vanderbilt University Hospital emergency department	Study involving recordings of Vanderbilt University Hospital emergency department inpatient admissions	Nik Hashim et al [32] Yingthawornsuk et al [39,40] Yingthawornsuk and Shiavi [41]
Cincinnati Children’s Hospital interview corpus	60 adolescents enrolled in a prospective study, 30 presenting to the emergency department with suicidal ideation and behaviors versus 30 controls presenting with orthopedic injuries	Scherer et al [15,22] Venek et al [23] Pestian et al [37]
DAIC^c^, 2014 [46]	Database of 621 recordings of distressed and nondistressed individuals diagnosed with anxiety, depression, and posttraumatic stress disorder; interviews were conducted in person and via an autonomous agent	Scherer et al [22]
AVEC^d^	Database of 292 audiovisual recordings of interviews with participants with depression conducted via an autonomous agent	Scherer et al [22]
Temuco data set	Database of recordings of interviews with 60 first-year university students from the Faculty of Health Sciences of the Autonomous University of Chile, Temuco	Figueroa Saavedra et al [29]
Washington DC^e^ Veterans Affairs Medical Center trial	Large ongoing prospective trial of veterans diagnosed with Gulf War syndrome	Belouali et al [28]

^a^DSM-IV: Diagnostic and Statistical Manual of Mental Disorders, Fourth Edition.

^b^nd: no date.

^c^DAIC: Distress Assessment Interview Corpus.

^d^AVEC: Audio-Visual Depression Corpus.

^e^DC: District of Columbia.

### What Are the Voice Signal Characteristics That Distinguish Elevated Suicide Risk From Other Cohorts?

Most studies (8/21, 38%) used frequency-based characteristics to differentiate participants at high risk of suicide from depressed and healthy cohorts, whereas 33% (7/21) of the studies used power spectral densities, 29% (6/21) used mel-frequency cepstral coefficients, 24% (5/21) used glottal cycle characteristics, and 14% (3/21) used timing patterns of speech. The highest median level of accuracy was attained using timing patterns of speech (85.5%), followed by power spectral densities (81.5%). Both the minimum and maximum levels of accuracy resulted from the use of power spectral densities (30.1% and 98.1%, respectively). In total, 19% (4/21) of the studies used vocal characteristics from a mixture of categories. For those studies that reported the levels of classification accuracy (15/21, 71%), median accuracies are reported in Table 3.

**Table 3:** Median classification accuracies of the voice signal characteristics selected in each study.

Primary feature	Studies	Accuracy (%), range	Accuracy (%), median
Frequency-based	Campbell [24] Francea et al [30] Ozdas et al [34] Beloualia et al [28] Pestiana et al [37] Figueroa Saavedra et al [29] Scherera et al [22] Sinha [26] Venek^a^ et al [23]	61.0-85.0	77.3
Power spectral densities	France et al [30] Nik Hashima et al [32] Keskinpala et al [31] Sanadi [25] Yingthawornsuk et al [39] Yingthawornsuk et al [40] Yingthawornsuk and Shiavi [41]	30.1-98.1	81.5
Mel-frequency cepstral coefficients	Belouali et al [28] Nik Hashim et al [32] Keskinpala et al [31] Ozdas et al [35] Subari et al [38] Yingthawornsuk et al [40]	60.0-90.0	78.3
Glottal cycle characteristics	Belouali et al [28] Ozdasa et al [35] Pestian et al [37] Scherera et al [15] Venek et al [23]	60.0-85.0	78.9
Timing patterns of speech	Nik Hashim et al [32] Nik Hashim et al [33] Scherer et al [22]	66.0-100.0	85.5

^a^Combined with other voice biometrics.

### A Comparison of 22 Measures for Identifying High Risk of Suicide

**Figure 3:**
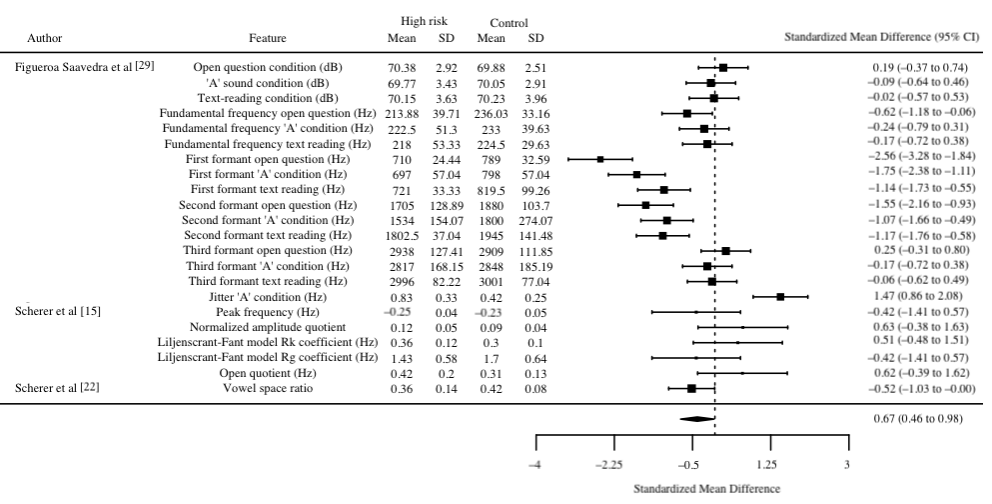
Random-effects meta-analysis forest plot illustrating 22 vocal characteristics in 3 studies.

A random-effects model meta-analysis was used to compare 22 different measures nested within 14% (3/21) of the publications. The included studies [15,22,29] involved 80 participants and 22 different vocal characteristics. The standardized mean difference for each vocal characteristic is summarized in the forest plot in Figure 3.

Positive standardized mean differences suggest higher levels of the vocal characteristic in the high–suicide-risk cohort, whereas negative standardized mean differences suggest that higher levels of the characteristic are found instead in the low-risk group.

A subgroup formed by the frequencies of the first and second formants applied to each of the 3 conditions reported by Figueroa Saavedra et al [29] was significant in differentiating between participants with and without elevated suicide risk. These significant negative standardized mean differences suggest that those at high risk of suicide feature lower vocal tract resonance frequencies, specifically in the lower formant regions rather than in the higher formant regions (ie, above the second formant of frequencies). Also of note were jitter values in the held “A” vowel condition in the study by Figueroa Saavedra et al [29]. Jitter in this condition yielded significant positive differentiation for participants with and without high suicide risk, suggesting that those at higher risk of suicide exhibited higher levels of roughness or hoarseness of articulation.

### What Are the Methodological Specifications Used?

Preprocessing is an important stage that occurs before the classification of vocal characteristics. This involves modifications to the voice signal to ensure greater precision in isolating its specific characteristics. Multimedia Appendix 5 illustrates an ideal preprocessing workflow.

The reviewed studies used a range of software for preprocessing and analysis of the vocal characteristics, including Microsound Editor [30,34,35,38,47] to identify and remove silence segments, MATLAB [22,23,25,26,30,39,48], COVAREP [22,23,37,49], and Praat [29,50] to facilitate subsequent analyses.

Most studies (11/21, 52%) [24,27,30,31,34-36,38-41] first converted the signal from analog to digital using a 16-bit recording at a sampling rate of 10 kHz. However, since 2010, recordings were sampled at higher rates. Venek et al [23] and Scherer et al [15,22], for instance, sampled speech at 16 kHz, whereas the remaining studies (19/21, 90%) [25,26,29,32,51] sampled it at 44.1 kHz.

All studies (21/21, 100%) then used a band-pass antialiasing filter to restrict the digital signal to a frequency range of 0 to 5000 Hz. Campbell [24] was the only author to analyze recordings sourced from telephone calls; thus, a band-pass filter restricting the frequency range to between 300 and 3000 Hz was automatically applied. After filtering, the signal was normalized [25,26,30,35,36,39-41] and detrended [25,30,39-41] to facilitate comparison between speakers and isolate the variable signal components, respectively.

Following these steps, the voice signal was differentiated between voiced and unvoiced types in 14% (3/21) of the studies. Subari et al [38] categorized voiced segments by the presence of cepstral peaks, Ozdas et al [34] differentiated between voiced and unvoiced signals using a discrete wavelet transform, whereas Sinha [26] adapted this approach to include 5 band-pass filters instead that selectively identified signal energies corresponding to each subband.

As noted, power spectral densities were investigated in several studies (6/21, 29%) [26,30,31,39-41]. Power spectral densities are derived from short-windowed segments of the voice signal. All studies (21/21, 100%) applied nonoverlapping Hamming windows to filter each 40- or 51.2-millisecond signal segment. A total of 10% (2/21) of the studies [38] used linear predictive coding applied to 15- and 25.6-millisecond segment durations to derive the first 3 formants and bandwidths.

Finally, most studies (7/21, 33%) used quadratic discriminant analysis to classify the voice signals of participants at high risk of suicide from other cohorts, and 57% (12/21) of the studies used either maximum likelihood, linear discriminant analysis, or support vector machines. As demonstrated in Table 4, the highest median level of accuracy was obtained using quadratic discriminant analysis. Both the minimum and maximum levels of accuracy were also recorded using quadratic discriminant analysis (21.4% and 100%, respectively). The median levels of classification accuracy are summarized in Table 4.

**Table 4:** Median accuracies achieved by classification algorithm.

Algorithm	Studies	Classification accuracy (%)	
		Range	Median
Maximum likelihood	Ozdas et al [34] Ozdas et al [35] Ozdas et al [36] Subari et al [38]	60.0-85.0	80.0
Linear discriminant analysis	France et al [30] Nik Hashim et al [51] Sanadi [25] Sinha [26]	30.1-98.1	79.7
Quadratic discriminant analysis	Nik Hashim et al [51] Keskinpala et al [31] Sanadi [25] Sinha [26] Yingthawornsuk et al [39] Yingthawornsuk et al [40] Yingthawornsuk and Shiavi [41]	21.4-100.0	85.4
Hierarchical mixed model	Scherer et al [15]	69.0-81.0	75.0
Support vector machine	Pestian et al [37] Scherer et al [15] Venek et al [23] Belouali et al [28]	61.0-85.0	75.9

### Risk of Bias and Quality Assessment

The risk of bias was assessed to explore the variability in the quality of the included publications. None of the included studies were at low risk of bias. In 43% (9/21) of the studies, the risk of bias was moderate [15,22,23,26,28-30,32,36-41], whereas in the remaining 57% (12/21), the risk of bias was assessed as high [24,25,27,31,33-35]. The main sources of bias were confounding factors, selection of participants, and selective reporting of results. The quality of evidence of most (18/21, 86%) of the included studies [15,22,23,27-37,39-41] was assessed with a rating of 3, whereas 14% (3/21) of the studies [17,24,52] were assessed at the lowest rating of 5.

## Discussion

### Principal Findings

A systematic review (1995-2021) was undertaken with 2 objectives in mind: to identify which vocal characteristics could accurately differentiate between individuals at high risk of suicide compared with those at lower risk and identify the methodological specifications that inform the derivation of each vocal characteristic and the classification accuracies that result.

A number of vocal characteristics were found to differentiate between high risk of suicide and comparison cohorts with a high level of accuracy. Of note were the median accuracies obtained using the timing patterns of speech (median accuracy 85.5%) and power spectral densities (median accuracy 81.5%). Furthermore, a random-effects meta-analysis that included 22 vocal characteristics from 14% (3/21) of the studies revealed that frequencies within the lower formants (1 and 2) and jitter provided significant standardized mean differences between high- and low–suicide-risk signals, suggesting that participants at high risk of suicide may have lower vocal tract resonance frequencies while speaking with greater roughness of speech.

These results are broadly consistent with several recent investigations that have also found significant increases in lower formant frequencies under stressful conditions, suggesting a reduction in articulatory clarity [17]; decreases in the quantity of speech among those at high risk of suicide [53]; and changes in jitter, a measure of cycle-to-cycle variation in the fundamental frequency that decreases under anxiety-producing conditions [17].

The findings of this study that mel-frequency cepstral coefficients characterize muscle tension and control of the vocal tract, a measure particularly sensitive to changes in stress, are also supported by previous research [54]. However, although increases in root mean squared amplitude or loudness have been found among speakers at high risk of suicide in more recent studies [55], the use of this variable was limited to only select studies reviewed (2/21, 10%) [30,37].

This review also aimed to identify the system specifications used to derive each vocal characteristic and their levels of accuracy. All studies (21/21, 100%) were found to adopt a similar workflow of preprocessing steps that broadly included (1) conversion from analog to digital signals, (2) band-pass filtering, (3) normalization and detrending, (4) differentiation between voiced and unvoiced signals, (5) removal of silent passages, and (6) signal segmentation before classification.

Although most studies (12/21, 57%) used band-pass filtering to remove frequencies >5000 Hz, only Campbell [24] used recordings sourced from telephone calls. As noted by the author, these sources automatically filter signals to between 300 and 3000 Hz. Although noise is reduced, this approach also removes the fundamental frequency from the signal signature but is known to overestimate the first formant frequency values by as much as 13% [56]. These may be important considerations for future studies that aim to source data from more novel settings such as telephone helplines or conversational agents.

Most studies (14/21, 67%) discussed the use of signal normalization to ensure a comparison between speakers. However, only in the study by Subari et al [38] was the effect on the overall accuracy of different forms of normalization investigated. Both a maximum likelihood–derived warping factor and normalization based on median third formant values were optimized based on the levels of classification accuracy. The authors noted that the formant-derived approach is preferable given its lower computational load and consideration of the sex of the participant. Normalization is a crucial consideration when the vocal tract of speakers can differ by as much as 7 cm [57].

Detrending was discussed in some studies (5/21, 24%). It is presumed that, by removing the mean signal, the authors sought to reveal the nonstationary signal components that might better differentiate one speaker from another. Although voice signals are known to be stationary only over short time frames (<40 ms) [58], several studies (8/21, 38%) used signal segmentation in excess of 50 milliseconds. The strategy of mean removal with potentially nonstationary signals risks dampening low-frequency sounds while attenuating high-frequency sounds and can also introduce secondary artifacts that may cloud ongoing analyses [58]. The preference in several studies (5/21, 24%) to detrend via discrete wavelet transform overcomes many of the aforementioned issues and appears well suited to the analysis of nonstationary signals at longer time frames of capture.

Given the differences in the frequency and amplitude spectra between voiced and unvoiced segments of speech, it is unsurprising that most of the reviewed publications (11/21, 52%) opted to differentiate between these signal types before classification. In total, 3 different approaches to voiced or unvoiced signal differentiation were used: approximation of voiced signals via the presence of cepstral peaks; frequency mapping via discrete wavelet transform using the highest-scale wavelets (2^5^) to categorize voiced signals; and selective band-pass filtering with frequencies >2500 Hz categorized as unvoiced, whereas signals between 320 and 2499 Hz were categorized as voiced. Further investigation is required to determine which approach best optimizes accuracies of classification; however, selective band-pass filtering has the advantage of not altering the signal in any way.

Regarding those studies that analyzed power spectral densities (9/21, 43%), nonoverlapping Hamming windows were the preferred approach to derive the short–time frame Fourier transform, converting the signal from the time to the frequency domain. This nonstandard approach has the effect of capturing frequencies at regular intervals corresponding to window width while introducing high frequencies as each window tapers into the next. The standard approach that incorporates overlapping windows, smoothing the effects at the tails, seems preferable to the nonstandard approach commonly used [59].

In only a minority of the reviewed studies (3/21, 14%) was supervised machine learning used (support vector machine). However, the highest median levels of accuracy were obtained not when these advanced forms of classification were used but rather when unsupervised quadratic discriminant analysis was used. Contrary to the much touted superiority of supervised machine learning methods, our findings suggest that higher levels of accuracy were instead obtained using less complex classification approaches. However, further investigation using more sophisticated approaches such as neural networks is clearly warranted.

In only the study by Scherer et al [15] was a mixed-effects model used. This approach might better capture the correlated structure of voice signal segments and better account for intraspeaker variance than other approaches.

### Future Directions and Implications for Practice

Suicide risk has been treated as static, stable, and invariant following initial assessment. Recent studies demonstrate that suicide risk can, in fact, change dramatically over time, suggesting that future studies might use insights from ecological momentary assessment [52,60]. Alternatively, future studies could adopt the approach of Campbell [24] by using trained personnel to assess the changing level of suicide risk across time within each recording as well as between recordings. Such approaches might better reflect the real-time change in suicidality, acknowledging that individuals frequently cycle in and out of risk.

In the reviewed literature, risk assessments were typically performed by coauthors (eg, Silverman in the study by Campbell [24] or Salomon in the study by Sinha [26]), an approach that may bias the objective assessment of risk. Future research might use multiple assessors of suicide risk where measures of interrater reliability can be analyzed and possible biases can be isolated and addressed.

Our analysis of the preprocessing workflows suggests that greater transparency regarding methodological considerations is urgently required. The reviewed publications were clearly aimed at an informed and knowledgeable engineering readership, and it was common to refer to complex methods using technical terms (eg, windowing). It would assist in reproducibility to understand preprocessing decisions in greater detail (ie, window type).

Certain vocal characteristics have been proven to more accurately differentiate between high and low risk of suicide. In particular, the timing patterns of speech and the vowel space occupied by different speaker articulations hold considerable promise. Also of note are insights derived from power spectral densities and frequency-related categories, underused methods such as the Liljencrants-Fant model of glottal flow, and mel-frequency cepstral coefficients. However, as demonstrated by Pestian et al [37] and Venek et al [23], the future of research in this field leans toward combining multiple features within high-powered machine learning algorithms such as support vector machine, although it should be noted that lower-powered approaches appeared in this review to yield greater levels of accuracy (ie, quadratic discriminant analysis).

The power of these advanced machine learning algorithms can only be used with an adequately powered sample. Cummins et al [14] called for greater collaboration between research teams to address this ongoing problem. An alternative approach might be to secure greater industry partnerships and look to novel settings with high call volumes such as telemental health. Given that a recent review found poor support for conventional suicide screening methods [10], there is a clear case for incorporating voice signal–informed analysis into existing telehealth and other e-services, in particular suicide helplines. These settings typically have large call volumes that increasingly feature elevated risk of suicide, particularly in the COVID-19 era. However, such collaborations also raise other ethical issues such as how best to safeguard callers’ rights to privacy and secure consent.

### Limitations

There are some limitations to this review. Of note was the lack of specificity in the definitions of high risk of suicide. In only 43% (9/21) of the studies analyzed, the high-risk cohorts were truly reflective of imminent risk of suicide. These studies used recordings of participants sourced from the Silverman database of suicide notes left by patients who had either attempted or completed suicide or, alternatively, from the Cincinnati Children’s Hospital interview corpus, where participants were recruited and interviewed immediately following presentation at emergency departments with acute suicidality. In the remaining studies (12/21, 57%), participants were assigned to the high-risk cohort based on cutoff scores on diverse psychometric tests, including the Beck Depression Inventory and Hamilton Depression Rating Scale. Several large-scale reviews [10,61-63] attest to the low precision and recall of suicide rating scales, suggesting that participants assigned to cohorts with high risk of suicide in these studies may also feature a proportion of false positives and, conversely, a proportion of false negatives in the control groups.

There was also a diversity of vocal characteristics trialed. Across the reviewed publications, it was challenging to find the same set of features replicated in other samples. It was more common to find a single characteristic combined with others to optimize discrimination between levels of suicide risk. This presented difficulties in determining which features might be reliably accurate across different settings.

Except for a notable large-scale multicenter trial [37], most of the reviewed studies (17/21, 81%) involved small samples, typically <60 participants, sometimes divided between 3 comparison groups [38,41]. As highlighted by Button et al [64], small-sample research is plagued by a number of issues, namely, reduced power, low reproducibility of results, and reductions in the likelihood that the results obtained reflect a true effect. Undoubtedly, these problems are amplified when the sample size of the comparison groups is <10, as was the case in several of the studies reviewed (8/21, 38%) [24,26,34-36,38,40,41]. Also of consideration were the high proportion of studies involving psychiatric inpatients (17/21, 81%) and the homogeneity of the databases from which participants were recruited, further limiting generalizability.

Also of note were the controlled conditions within which the trials took place. It was common for participants to be invited into a room away from extraneous noise and asked to read prescripted text such as the rainbow passage (eg, Yingthawornsuk et al [39]) or to articulate prolonged vowel sounds (eg, Scherer et al [22]). Although these research protocols increase the likelihood of uncovering candidate vocal characteristics, they also reduce the generalizability of the research findings to other settings, particularly to telephony and other novel eHealth-based applications where these controls are impractical to implement and noise is the rule rather than the exception. Except for the study by Campbell [24], no studies sourced participants from these more ecologically valid settings.

There was a paucity of detail relating to specific preprocessing elements supported by the high risk of bias prevailing among the reviewed studies. One of the cardinal requirements for cumulative science is that methodologies are replicable [65]. Publications are increasingly restricting the number of allowable words. Prospective authors might be tempted to limit descriptions of the methodology in favor of the results and discussion. However, most publications also allow for appendixes that can provide supplementary information relating to the methodology.

However, there were also notable strengths to this review. This review has expanded upon the findings of Cummins et al [14] and Homan et al [16] in important ways. We have updated the research findings to 2021, and based on the accuracy of discrimination between levels of suicide risk, we were able to identify a number of promising candidate vocal characteristics that warrant further investigation. We were also able to identify and discuss a number of preprocessing steps used before the classification of voice signals.

### Conclusions

The data indicate that several characteristics successfully differentiate between individuals at high and low risk of suicide. An analysis of power spectral density subbands yielded high accuracies of discrimination between comparison groups (eg, 90.3% accuracy in the study by Yingthawornsuk et al [40]); however, the studies that used power spectral densities disagreed on whether the lower- or higher-frequency subbands were of key importance and also disagreed on the need for single or combined feature analyses. Second, several studies (4/21, 19%) found higher formant frequencies and a narrowing of bandwidth among those at elevated risk of suicide [22,23,29,30]. Higher levels of predictive accuracy were found when formant features were combined with other features (eg, 80% accuracy in the study by France et al [30]). Third, Nik Hashim et al [32,51] and Scherer et al [22] found that the timing patterns of speech in speakers at elevated risk of suicide differed in a number of important ways from those of speakers at low risk of suicide. In particular, pauses were protracted, whereas certain vowel sounds were held for longer periods among those at elevated risk of suicide. Fourth, both the study by Anunvrapong and Yingthawornthuk [27] and the study by Ozdas et al [35] found that the analysis of mel-frequency cepstral coefficients—which attempts to mimic the energy spectrum of human hearing—successfully differentiated between speakers at high and low risk of suicide. However, a reduced filter bank (first 4 frequencies) yielded greater accuracies. Finally, both Scherer et al [22] and Venek et al [23] found that certain coefficients of the Liljencrants-Fant model of glottal flow significantly differentiated between high and low risk of suicide, suggesting that those at high risk of suicide often speak in breathier tones. This was particularly apparent among adolescents.

Although this systematic review revealed a number of limitations in the current literature in this field, the level of accuracy achieved is promising, suggesting that future research, particularly in more novel areas of telemental health, holds considerable promise for the detection and prevention of suicide in the community.

## Appendix

Multimedia Appendix 1 PRISMA (Preferred Reporting Items for Systematic Reviews and Meta-Analyses) checklist.

Multimedia Appendix 2 Representative search strategies. STFT: short–time frame Fourier transform.

Multimedia Appendix 3 Risk-of-bias assessments.

Multimedia Appendix 4 Review data.

Multimedia Appendix 5 Ideal preprocessing workflow.

## Abbreviations

PRISMA: Preferred Reporting Items for Systematic Reviews and Meta-Analyses
