# Supplementary figures and images for: Detection of Suicide Risk Using Vocal Characteristics: Systematic Review
Source: JMIR Biomed Eng. 2022 Dec 22;7(2):e42386. doi: 10.2196/42386 (PMC11041425; doi:10.2196/42386)

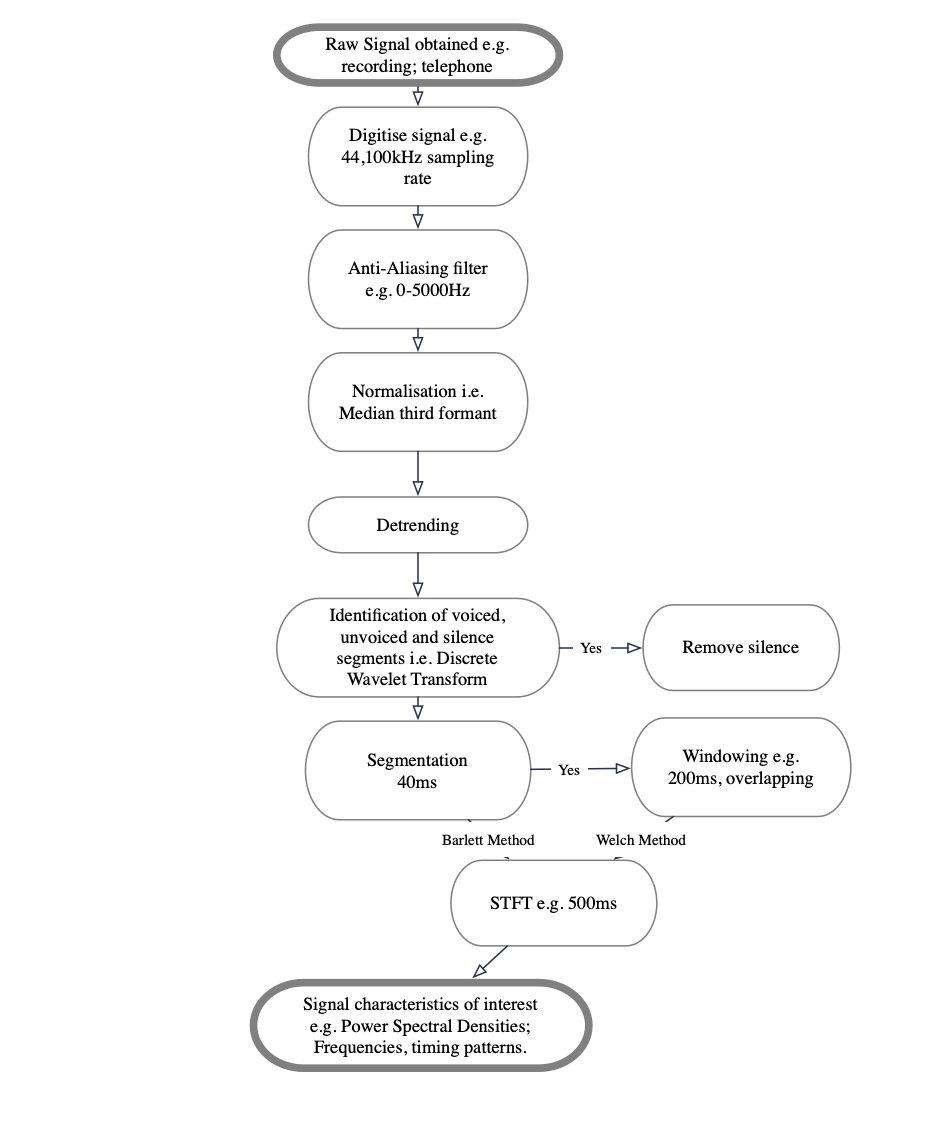

Supplement: Multimedia Appendix 2 [file biomedeng_v7i2e42386_app2.png]
