# Supplementary material for: Detection of Suicide Risk Using Vocal Characteristics: Systematic Review
Source: JMIR Biomed Eng. 2022 Dec 22;7(2):e42386. doi: 10.2196/42386 (PMC11041425; doi:10.2196/42386)
Supplement: Multimedia Appendix 3 [file biomedeng_v7i2e42386_app3.docx]

|  | **Bias due to confounding** | **Bias in selection of participants** | **Bias in classification of exposures** | **Bias due to deviations from intended exposures** | **Bias due to missing data** | **Bias in measurement of the outcome** | **Bias in selection of the reported result** |  | **Study level RoB** |
| --- | --- | --- | --- | --- | --- | --- | --- | --- | --- |
| Anunvrapong (2014) |  |  |  |  |  |  |  |  |  |
| Belouali (2021) |  |  |  |  |  |  |  |  |  |
| Campbell (1995) |  |  |  |  |  |  |  |  |  |
| France(2000) |  |  |  |  |  |  |  |  |  |
| Keskinpala (2007) |  |  |  |  |  |  |  |  |  |
| Ozdas (2000) |  |  |  |  |  |  |  |  |  |
| Ozdas (2004a) |  |  |  |  |  |  |  |  |  |
| Ozdas (2004b) |  |  |  |  |  |  |  |  |  |
| Pestian (2017) |  |  |  |  |  |  |  |  |  |
| Saavedra (2020) |  |  |  |  |  |  |  |  |  |
| Sanadi (2011) |  |  |  |  |  |  |  |  |  |
| Scherer (2013) |  |  |  |  |  |  |  |  |  |
| Scherer (2015) |  |  |  |  |  |  |  |  |  |
| Sinha (2013) |  |  |  |  |  |  |  |  |  |
| Subari (2010) |  |  |  |  |  |  |  |  |  |
| Venek (2017) |  |  |  |  |  |  |  |  |  |
| Wahidah (2012) |  |  |  |  |  |  |  |  |  |
| Wahidah(2015) |  |  |  |  |  |  |  |  |  |
| Yingthawornsuk (2006) |  |  |  |  |  |  |  |  |  |
| Yingthawornsuk (2007a) |  |  |  |  |  |  |  |  |  |
| Yingthawornsuk (2008) |  |  |  |  |  |  |  |  |  |
|  |  |  |  |  |  |  |  |  |  |
| **Item level RoB** |  |  |  |  |  |  |  |  |  |
