# Supplementary material for: Detection of Suicide Risk Using Vocal Characteristics: Systematic Review
Source: JMIR Biomed Eng. 2022 Dec 22;7(2):e42386. doi: 10.2196/42386 (PMC11041425; doi:10.2196/42386)
Supplement: Multimedia Appendix 5 [file biomedeng_v7i2e42386_app5.docx]

Multimedia Appendix 5

Medline search – Ovid interface.

1^st^ January 1995 to 1^st^ November 2021

Exp. Suicide/ NOT assisted

Exp. Self-injurious behaviour/ NOT self-mutilation

Suicid*.ti,ab,kw

Self-harm. ti,ab,kw

Suicide ADJ4 risk. ti,ab,kw

OR 1-5

Exp. Speech/

Exp. Voice quality/

Voice. ti,ab,kw

Waveform. ti,ab,kw

Vocal. ti,ab,kw

Signal. ti,ab,kw

Paralinguistic. ti,ab,kw

OR 7-14

Exp. Algorithm/ NOT latent class analysis

“deep learning”. ti,ab,kw

“machine learning”. ti,ab,kw

OR 16-18

Prosodic. ti,ab,kw

Formant. ti,ab,kw

Glott*. ti,ab,kw

Spectral. ti,ab,kw

Fourier. ti,ab,kw

Jitter. ti,ab,kw

“fundamental frequency”. ti,ab,kw

OR 19-25

6 AND 14 AND (18 OR 26)

Limit to human(s)

*=truncation, exp=explode, ADJ4=adjacent to keyword within 4 terms in either direction, .ti,ab,kw=search the title, abstract and keyword fields only

Final Web of Science search.

1^st^ January 1995 to 1^st^ November 2021

ALL=Suicid* NOT ALL=assisted

ALL=self harm

ALL=suicid* NEAR/4 risk

OR #1-#3

ALL=voice

ALL=waveform

ALL=vocal

ALL=signal

ALL=paralinguistic

OR #5-#9

ALL=Artificial Intelligence

ALL=Deep learning

ALL=Machine learning

ALL=Algorithm

OR #11-#14

ALL=prosocid

ALL=formant

ALL=glott*

ALL=spectral

ALL=fourier

ALL=jitter

ALL=fundamental frequency

OR #16=#22

#4 AND #10 AND (#15 OR #23)

*Truncation, NEAR/4=Adjacent to keyword within 4 terms either side, ALL=All fields
